# Supplementary material for: Open Soil Spectral Library (OSSL): Building reproducible soil calibration models through open development and community engagement
Source: PLoS One. 2025 Jan 13;20(1):e0296545. doi: 10.1371/journal.pone.0296545 (PMC11730021; doi:10.1371/journal.pone.0296545)
Supplement: S1 Table — (PDF) [file pone.0296545.s001.pdf]

Supporting Information of *Open Soil Spectral Library (OSSL): Building reproducible soil calibration models through open development and community engagement*

José L. Safanelli[1], Tomislav Hengl[2], Leandro L. Parente[2], Robert Minarik[2], Dellena E. Bloom[3], Katherine Todd-Brown[3], Asa Gholizadeh[4], Wanderson de Sousa Mendes[5], Jonathan Sanderman[1*]

1 Woodwell Climate Research Center, Falmouth, MA, USA

2 OpenGeoHub foundation, Wageningen, the Netherlands

3 University of Florida, Gainesville, FL, USA

4 Czech University of Life Sciences Prague, Czech Republic

5 The Food and Agriculture Organization of the United Nations, Rome, Italy

* Corresponding author. E-mail: jsanderman@woodwellclimate.org

S1 Table. Summary statistics of soil properties grouped by spectral region imported into the Open Soil Spectral Library (OSSL): Soil property descriptions are supplied in Table 2.

Soil property	Subset	Spectra	n	min.	Q1	median	Q3	max.
acidity_usda.a795_cmolc.kg	OSSL	MIR	30061	0.00	3.50	7.07	13.80	282.72
acidity_usda.a795_cmolc.kg	OSSL	NIR	1576	0.05	3.85	8.05	13.88	157.00
acidity_usda.a795_cmolc.kg	OSSL	VisNIR	1511	0.00	0.20	0.90	2.60	25.50
aggstb_usda.a1_w.pct	OSSL	MIR	3218	0.00	9.00	28.00	63.00	106.00
aggstb_usda.a1_w.pct	OSSL	NIR	197	0.00	12.00	40.00	70.00	100.00
aggstb_usda.a1_w.pct	OSSL	VisNIR	0	0.00	0.00	0.00	0.00	0.00
al.dith_usda.a65_w.pct	OSSL	MIR	31135	0.00	0.05	0.11	0.21	7.70
al.dith_usda.a65_w.pct	OSSL	NIR	1773	0.00	0.04	0.10	0.18	2.28
al.dith_usda.a65_w.pct	OSSL	VisNIR	0	0.00	0.00	0.00	0.00	0.00
al.ext_usda.a1056_mg.kg	OSSL	MIR	3773	0.00	418.57	746.70	1154.47	6748.01

Continuation of S1 Table

Soil property	Subset	Spectra	n	min.	Q1	median	Q3	max.
al.ext_usda.a1056_mg.kg	OSSL	NIR	76	5.72	588.95	865.62	1040.48	2238.66
al.ext_usda.a1056_mg.kg	OSSL	VisNIR	0	0.00	0.00	0.00	0.00	0.00
al.ext_usda.a69_cmolc.kg	OSSL	MIR	15710	0.00	0.28	1.01	2.87	63.01
al.ext_usda.a69_cmolc.kg	OSSL	NIR	716	0.00	0.33	1.02	2.55	16.44
al.ext_usda.a69_cmolc.kg	OSSL	VisNIR	1541	0.00	0.00	0.70	2.10	25.30
al.ox_usda.a59_w.pct	OSSL	MIR	28260	0.00	0.06	0.12	0.24	8.80
al.ox_usda.a59_w.pct	OSSL	NIR	1354	0.00	0.07	0.12	0.21	4.00
al.ox_usda.a59_w.pct	OSSL	VisNIR	0	0.00	0.00	0.00	0.00	0.00
awc.33.1500kPa_usda.c80_w.frac	OSSL	MIR	16175	0.00	0.11	0.14	0.19	0.78
awc.33.1500kPa_usda.c80_w.frac	OSSL	NIR	931	0.00	0.11	0.15	0.19	0.51
awc.33.1500kPa_usda.c80_w.frac	OSSL	VisNIR	0	0.00	0.00	0.00	0.00	0.00
b.ext_mel3_mg.kg	OSSL	MIR	2093	0.00	0.00	0.12	0.31	55.09
b.ext_mel3_mg.kg	OSSL	NIR	0	0.00	0.00	0.00	0.00	0.00
b.ext_mel3_mg.kg	OSSL	VisNIR	0	0.00	0.00	0.00	0.00	0.00
bd_usda.a4_g.cm3	OSSL	MIR	41484	0.01	0.92	1.25	1.45	8.10
bd_usda.a4_g.cm3	OSSL	NIR	1085	0.20	1.18	1.32	1.46	2.03
bd_usda.a4_g.cm3	OSSL	VisNIR	20751	0.06	0.90	1.20	1.41	1.98
c.tot_usda.a622_w.pct	OSSL	MIR	80698	0.00	0.58	1.58	4.29	78.45
c.tot_usda.a622_w.pct	OSSL	NIR	1976	0.02	0.82	1.61	3.00	53.88
c.tot_usda.a622_w.pct	OSSL	VisNIR	19807	0.01	0.68	2.05	19.71	78.45
ca.ext_usda.a1059_mg.kg	OSSL	MIR	3773	0.00	325.00	879.00	2977.53	44723.96
ca.ext_usda.a1059_mg.kg	OSSL	NIR	76	38.72	524.33	2506.14	3550.48	18563.27
ca.ext_usda.a1059_mg.kg	OSSL	VisNIR	0	0.00	0.00	0.00	0.00	0.00
ca.ext_usda.a722_cmolc.kg	OSSL	MIR	57085	0.00	3.00	11.35	27.12	410.41
ca.ext_usda.a722_cmolc.kg	OSSL	NIR	1976	0.00	2.58	10.52	23.17	363.64
ca.ext_usda.a722_cmolc.kg	OSSL	VisNIR	3723	0.00	0.40	3.80	15.20	168.20
caco3_usda.a54_w.pct	OSSL	MIR	29225	0.00	0.24	1.57	10.82	101.37
caco3_usda.a54_w.pct	OSSL	NIR	665	0.01	0.29	1.91	8.23	89.03
caco3_usda.a54_w.pct	OSSL	VisNIR	51094	0.00	0.00	0.10	2.10	99.70

Continuation of S1 Table

Soil property	Subset	Spectra	n	min.	Q1	median	Q3	max.
cec_usda.a723_cmolc.kg	OSSL	MIR	57651	0.00	7.78	15.33	24.66	584.59
cec_usda.a723_cmolc.kg	OSSL	NIR	1976	0.13	7.98	14.40	22.09	190.11
cec_usda.a723_cmolc.kg	OSSL	VisNIR	22687	0.00	6.80	12.30	20.50	234.00
cf_usda.c236_w.pct	OSSL	MIR	56470	0.00	0.00	0.25	7.00	100.00
cf_usda.c236_w.pct	OSSL	NIR	1969	0.00	0.04	1.00	14.00	93.00
cf_usda.c236_w.pct	OSSL	VisNIR	23237	0.00	6.00	11.00	20.00	90.00
clay.tot_usda.a334_w.pct	OSSL	MIR	57268	0.00	10.30	21.77	33.99	100.00
clay.tot_usda.a334_w.pct	OSSL	NIR	1976	0.00	8.75	17.84	27.75	76.97
clay.tot_usda.a334_w.pct	OSSL	VisNIR	27145	0.00	8.00	17.20	28.00	96.80
cu.ext_usda.a1063_mg.kg	OSSL	MIR	3772	0.00	0.45	1.20	2.66	77.82
cu.ext_usda.a1063_mg.kg	OSSL	NIR	76	0.05	0.89	1.95	3.09	10.90
cu.ext_usda.a1063_mg.kg	OSSL	VisNIR	0	0.00	0.00	0.00	0.00	0.00
ec_usda.a364_ds.m	OSSL	MIR	34040	0.00	0.10	0.25	0.87	313.13
ec_usda.a364_ds.m	OSSL	NIR	942	0.01	0.14	0.23	0.46	81.93
ec_usda.a364_ds.m	OSSL	VisNIR	21833	0.00	0.10	0.17	0.28	9.69
fe.dith_usda.a66_w.pct	OSSL	MIR	31138	0.00	0.33	0.82	1.56	28.44
fe.dith_usda.a66_w.pct	OSSL	NIR	1773	0.00	0.41	0.83	1.40	9.81
fe.dith_usda.a66_w.pct	OSSL	VisNIR	0	0.00	0.00	0.00	0.00	0.00
fe.ext_usda.a1064_mg.kg	OSSL	MIR	3773	0.00	59.35	94.14	151.10	2708.08
fe.ext_usda.a1064_mg.kg	OSSL	NIR	76	16.63	62.27	90.28	150.64	303.84
fe.ext_usda.a1064_mg.kg	OSSL	VisNIR	0	0.00	0.00	0.00	0.00	0.00
fe.ox_usda.a60_w.pct	OSSL	MIR	28259	0.00	0.09	0.24	0.54	22.27
fe.ox_usda.a60_w.pct	OSSL	NIR	1354	0.00	0.10	0.21	0.45	3.42
fe.ox_usda.a60_w.pct	OSSL	VisNIR	0	0.00	0.00	0.00	0.00	0.00
k.ext_usda.a1065_mg.kg	OSSL	MIR	3772	0.00	52.47	102.89	200.02	5047.00
k.ext_usda.a1065_mg.kg	OSSL	NIR	76	0.00	82.01	147.45	256.33	730.19
k.ext_usda.a1065_mg.kg	OSSL	VisNIR	0	0.00	0.00	0.00	0.00	0.00
k.ext_usda.a725_cmolc.kg	OSSL	MIR	57674	0.00	0.15	0.35	0.73	37.56
k.ext_usda.a725_cmolc.kg	OSSL	NIR	1976	0.00	0.15	0.35	0.70	11.25

Continuation of S1 Table

Soil property	Subset	Spectra	n	min.	Q1	median	Q3	max.
k.ext_usda.a725_cmolc.kg	OSSL	VisNIR	44489	0.00	0.33	0.67	1.21	51.31
mg.ext_usda.a1066_mg.kg	OSSL	MIR	3773	0.00	75.33	182.00	532.77	5608.60
mg.ext_usda.a1066_mg.kg	OSSL	NIR	76	4.53	97.13	346.45	693.75	2153.54
mg.ext_usda.a1066_mg.kg	OSSL	VisNIR	0	0.00	0.00	0.00	0.00	0.00
mg.ext_usda.a724_cmolc.kg	OSSL	MIR	57093	0.00	0.88	2.72	6.40	172.64
mg.ext_usda.a724_cmolc.kg	OSSL	NIR	1976	0.00	0.68	2.01	4.34	82.14
mg.ext_usda.a724_cmolc.kg	OSSL	VisNIR	3731	0.00	0.20	1.10	3.40	68.00
mn.ext_usda.a1067_mg.kg	OSSL	MIR	3772	0.00	18.10	55.70	120.52	686.60
mn.ext_usda.a1067_mg.kg	OSSL	NIR	76	0.12	39.12	63.46	126.74	334.24
mn.ext_usda.a1067_mg.kg	OSSL	VisNIR	0	0.00	0.00	0.00	0.00	0.00
mn.ext_usda.a70_mg.kg	OSSL	MIR	14166	0.00	0.17	1.03	4.41	9787.33
mn.ext_usda.a70_mg.kg	OSSL	NIR	716	0.00	0.17	0.86	2.86	143.37
mn.ext_usda.a70_mg.kg	OSSL	VisNIR	0	0.00	0.00	0.00	0.00	0.00
n.tot_usda.a623_w.pct	OSSL	MIR	81282	0.00	0.05	0.12	0.29	41.90
n.tot_usda.a623_w.pct	OSSL	NIR	1976	0.00	0.07	0.13	0.22	3.02
n.tot_usda.a623_w.pct	OSSL	VisNIR	60570	0.00	0.11	0.18	0.36	4.90
na.ext_usda.a1068_mg.kg	OSSL	MIR	3616	0.00	17.10	32.04	58.29	31800.00
na.ext_usda.a1068_mg.kg	OSSL	NIR	76	3.29	7.27	14.05	56.84	1739.24
na.ext_usda.a1068_mg.kg	OSSL	VisNIR	0	0.00	0.00	0.00	0.00	0.00
na.ext_usda.a726_cmolc.kg	OSSL	MIR	57075	0.00	0.00	0.00	0.22	868.36
na.ext_usda.a726_cmolc.kg	OSSL	NIR	1976	0.00	0.00	0.00	0.05	202.96
na.ext_usda.a726_cmolc.kg	OSSL	VisNIR	3715	0.00	0.00	0.10	0.30	31.60
oc_usda.c729_w.pct	OSSL	MIR	82573	0.00	0.38	1.13	3.52	78.45
oc_usda.c729_w.pct	OSSL	NIR	1974	0.02	0.65	1.39	2.71	53.88
oc_usda.c729_w.pct	OSSL	VisNIR	64211	0.00	0.98	1.88	4.34	78.45
p.ext_usda.a1070_mg.kg	OSSL	MIR	27690	0.00	2.86	10.47	34.99	24425.23
p.ext_usda.a1070_mg.kg	OSSL	NIR	754	0.00	3.59	12.68	36.12	1358.90
p.ext_usda.a1070_mg.kg	OSSL	VisNIR	0	0.00	0.00	0.00	0.00	0.00
p.ext_usda.a270_mg.kg	OSSL	MIR	7282	0.00	1.08	4.94	18.94	1436.68

Continuation of S1 Table

Soil property	Subset	Spectra	n	min.	Q1	median	Q3	max.
p.ext_usda.a270_mg.kg	OSSL	NIR	266	0.00	2.83	8.93	21.59	320.35
p.ext_usda.a270_mg.kg	OSSL	VisNIR	0	0.00	0.00	0.00	0.00	0.00
p.ext_usda.a274_mg.kg	OSSL	MIR	16084	0.00	1.82	5.82	17.00	686.28
p.ext_usda.a274_mg.kg	OSSL	NIR	281	0.00	2.05	4.27	12.24	623.13
p.ext_usda.a274_mg.kg	OSSL	VisNIR	40764	0.00	11.10	23.10	43.40	1366.40
ph.cacl2_usda.a481_index	OSSL	MIR	53819	2.14	4.78	5.73	7.23	10.68
ph.cacl2_usda.a481_index	OSSL	NIR	1976	3.23	4.64	5.58	7.04	9.46
ph.cacl2_usda.a481_index	OSSL	VisNIR	40848	2.57	4.40	5.70	7.10	10.00
ph.h2o_usda.a268_index	OSSL	MIR	59997	1.97	5.33	6.23	7.56	10.70
ph.h2o_usda.a268_index	OSSL	NIR	1976	3.69	5.23	6.17	7.40	9.52
ph.h2o_usda.a268_index	OSSL	VisNIR	44590	3.00	4.97	6.10	7.45	10.50
s.tot_usda.a624_w.pct	OSSL	MIR	76592	0.00	0.00	0.02	0.05	25.24
s.tot_usda.a624_w.pct	OSSL	NIR	1976	0.00	0.00	0.01	0.03	18.38
s.tot_usda.a624_w.pct	OSSL	VisNIR	19807	0.00	0.00	0.01	0.04	13.12
sand.tot_usda.c60_w.pct	OSSL	MIR	57162	0.00	12.20	33.00	60.90	100.00
sand.tot_usda.c60_w.pct	OSSL	NIR	1976	0.30	17.80	39.20	64.62	100.00
sand.tot_usda.c60_w.pct	OSSL	VisNIR	27086	0.00	17.00	37.00	60.00	100.00
silt.tot_usda.c62_w.pct	OSSL	MIR	57215	0.00	20.80	37.10	53.00	256.00
silt.tot_usda.c62_w.pct	OSSL	NIR	1976	0.00	22.70	38.10	52.60	87.90
silt.tot_usda.c62_w.pct	OSSL	VisNIR	27139	0.00	22.00	37.00	50.00	256.00
wr.1500kPa_usda.a417_w.pct	OSSL	MIR	41345	0.02	6.91	11.59	17.14	244.23
wr.1500kPa_usda.a417_w.pct	OSSL	NIR	1951	0.08	6.36	10.45	14.84	96.14
wr.1500kPa_usda.a417_w.pct	OSSL	VisNIR	971	0.10	12.90	21.90	29.55	56.40
wr.33kPa_usda.a415_w.pct	OSSL	MIR	19459	0.26	17.81	23.68	29.96	2124.87
wr.33kPa_usda.a415_w.pct	OSSL	NIR	1032	1.00	18.34	23.96	29.66	187.65
wr.33kPa_usda.a415_w.pct	OSSL	VisNIR	923	2.30	22.90	31.40	41.50	71.40
zn.ext_usda.a1073_mg.kg	OSSL	MIR	3760	0.00	0.56	1.04	1.87	314.72
zn.ext_usda.a1073_mg.kg	OSSL	NIR	76	0.00	0.84	1.92	3.65	35.95
zn.ext_usda.a1073_mg.kg	OSSL	VisNIR	0	0.00	0.00	0.00	0.00	0.00
