# Supplementary material for: Open Soil Spectral Library (OSSL): Building reproducible soil calibration models through open development and community engagement
Source: PLoS One. 2025 Jan 13;20(1):e0296545. doi: 10.1371/journal.pone.0296545 (PMC11730021; doi:10.1371/journal.pone.0296545)
Supplement: S2 Table — Note: Except for clay.tot_usda.a334_w.pct. silt.tot_usda.c62_w.pct, sand.tot_usda.c60_w.pct, ph.h2o_usda.a268_index, and ph.cacl2_usda.a481_index, all the other soil properties’ metrics are reported in the natural logarithm space (with offset = 1, log1p() R function). (PDF) [file pone.0296545.s002.pdf]

Supporting Information of *Open Soil Spectral Library (OSSL): Building reproducible soil calibration models through open development and community engagement*

José L. Safanelli[1], Tomislav Hengl[2], Leandro L. Parente[2], Robert Minarik[2], Dellena E. Bloom[3], Katherine Todd-Brown[3], Asa Gholizadeh[4], Wanderson de Sousa Mendes[5], Jonathan Sanderman[1*]

1 Woodwell Climate Research Center, Falmouth, MA, USA

2 OpenGeoHub foundation, Wageningen, the Netherlands

3 University of Florida, Gainesville, FL, USA

4 Czech University of Life Sciences Prague, Czech Republic

5 The Food and Agriculture Organization of the United Nations, Rome, Italy

* Corresponding author. E-mail: jsanderman@woodwellclimate.org

S5 Table. Goodness-of-fit metrics from 10-fold cross-validation with refitting, calculated across different soil properties and model types: Root mean squared error (RMSE), mean error (bias), coefficient of determination (R^2), Lin's concordance correlation coefficient (CCC), ratio of performance to the interquartile range (RPIQ). Note: Except for `clay.tot_usda.a334_w.pctsilt.tot_usda.c62_w.pct`, `sand.tot_usda.c60_w.pct`, `ph.h2o_usda.a268_index`, and `ph.cacl2_usda.a481_index`, all the other soil properties' metrics are reported in the natural logarithm space (with offset = 1, `log1p()` R function).

Soil property	Model	RMSE	bias	R^2	CCC	RPIQ
acidity_usda.a795_cmolc.kg	mir_cubist_kssl_na_v1.2	0.26	-0.01	0.93	0.96	4.38
acidity_usda.a795_cmolc.kg	mir_cubist_ossl_na_v1.2	0.27	-0.01	0.93	0.96	4.36
acidity_usda.a795_cmolc.kg	nir.neospectra_cubist_ossl_na_v1.2	0.51	-0.01	0.65	0.78	2.22
acidity_usda.a795_cmolc.kg	visnir_cubist_ossl_na_v1.2	0.42	0.02	0.65	0.77	2.59
aggstb_usda.a1_w.pct	mir_cubist_kssl_na_v1.2	0.64	-0.04	0.69	0.80	2.92

Continuation of S5 Table

Soil property	Model	RMSE	bias	R^2	CCC	RPIQ
aggstb_usda.a1_w.pct	mir_cubist_ossl_na_v1.2	0.64	-0.04	0.69	0.80	2.91
al.dith_usda.a65_w.pct	mir_cubist_kssl_na_v1.2	0.06	0.00	0.91	0.95	2.70
al.dith_usda.a65_w.pct	mir_cubist_ossl_na_v1.2	0.06	0.00	0.91	0.95	2.72
al.dith_usda.a65_w.pct	nir.neospectra_cubist_ossl_na_v1.2	0.09	0.00	0.74	0.84	1.49
al.ext_usda.a1056_mg.kg	mir_cubist_kssl_na_v1.2	0.52	-0.01	0.92	0.96	2.90
al.ext_usda.a1056_mg.kg	mir_cubist_ossl_na_v1.2	0.38	-0.02	0.92	0.96	2.69
al.ext_usda.a69_cmolc.kg	mir_cubist_kssl_na_v1.2	0.23	-0.00	0.90	0.95	4.80
al.ext_usda.a69_cmolc.kg	mir_cubist_ossl_na_v1.2	0.23	-0.00	0.90	0.94	4.74
al.ext_usda.a69_cmolc.kg	nir.neospectra_cubist_ossl_na_v1.2	0.45	0.03	0.54	0.70	2.16
al.ext_usda.a69_cmolc.kg	visnir_cubist_ossl_na_v1.2	0.43	0.02	0.63	0.76	2.66
al.ox_usda.a59_w.pct	mir_cubist_kssl_na_v1.2	0.06	0.00	0.94	0.97	2.60
al.ox_usda.a59_w.pct	mir_cubist_ossl_na_v1.2	0.06	0.00	0.94	0.97	2.60
al.ox_usda.a59_w.pct	nir.neospectra_cubist_ossl_na_v1.2	0.10	0.01	0.77	0.85	1.17
awc.33.1500kPa_usda.c80_w.frac	mir_cubist_kssl_na_v1.2	0.04	0.00	0.48	0.61	1.57
awc.33.1500kPa_usda.c80_w.frac	mir_cubist_ossl_na_v1.2	0.04	0.00	0.48	0.61	1.58
awc.33.1500kPa_usda.c80_w.frac	nir.neospectra_cubist_ossl_na_v1.2	0.05	0.00	0.17	0.31	1.37
b.ext_mel3_mg.kg	mir_cubist_ossl_na_v1.2	0.17	0.01	0.80	0.88	1.65
bd_usda.a4_g.cm3	mir_cubist_kssl_na_v1.2	0.12	-0.01	0.75	0.86	2.03
bd_usda.a4_g.cm3	mir_cubist_ossl_na_v1.2	0.12	-0.01	0.75	0.86	2.04
bd_usda.a4_g.cm3	nir.neospectra_cubist_ossl_na_v1.2	0.08	-0.01	0.45	0.62	1.46
bd_usda.a4_g.cm3	visnir_cubist_kssl_na_v1.2	0.14	-0.01	0.66	0.79	1.78
bd_usda.a4_g.cm3	visnir_cubist_ossl_na_v1.2	0.13	-0.01	0.67	0.79	1.79
c.tot_usda.a622_w.pct	mir_cubist_kssl_na_v1.2	0.10	0.00	0.99	1.00	12.25
c.tot_usda.a622_w.pct	mir_cubist_ossl_na_v1.2	0.10	0.00	0.99	1.00	11.76
c.tot_usda.a622_w.pct	nir.neospectra_cubist_ossl_na_v1.2	0.27	-0.01	0.77	0.87	2.89
c.tot_usda.a622_w.pct	visnir_cubist_kssl_na_v1.2	0.24	-0.01	0.97	0.98	10.54
c.tot_usda.a622_w.pct	visnir_cubist_ossl_na_v1.2	0.23	-0.01	0.97	0.98	10.91
ca.ext_usda.a1059_mg.kg	mir_cubist_kssl_na_v1.2	0.43	-0.02	0.94	0.97	4.78
ca.ext_usda.a1059_mg.kg	mir_cubist_ossl_na_v1.2	0.41	-0.02	0.94	0.97	5.36

Continuation of S5 Table

Soil property	Model	RMSE	bias	R^2	CCC	RPIQ
ca.ext_usda.a722_cmolc.kg	mir_cubist_kssl_na_v1.2	0.27	-0.01	0.96	0.98	7.03
ca.ext_usda.a722_cmolc.kg	mir_cubist_ossl_na_v1.2	0.28	-0.01	0.95	0.98	7.01
ca.ext_usda.a722_cmolc.kg	nir.neospectra_cubist_ossl_na_v1.2	0.58	-0.02	0.80	0.89	3.30
ca.ext_usda.a722_cmolc.kg	visnir_cubist_ossl_na_v1.2	0.51	-0.01	0.85	0.92	4.85
caco3_usda.a54_w.pct	mir_cubist_kssl_na_v1.2	0.18	-0.00	0.98	0.99	12.58
caco3_usda.a54_w.pct	mir_cubist_ossl_na_v1.2	0.20	-0.00	0.97	0.99	11.23
caco3_usda.a54_w.pct	nir.neospectra_cubist_ossl_na_v1.2	0.65	-0.02	0.67	0.80	3.06
caco3_usda.a54_w.pct	visnir_cubist_kssl_na_v1.2	0.39	0.02	0.87	0.93	2.91
caco3_usda.a54_w.pct	visnir_cubist_ossl_na_v1.2	0.36	0.01	0.91	0.95	3.14
cec_usda.a723_cmolc.kg	mir_cubist_kssl_na_v1.2	0.16	-0.00	0.97	0.98	6.68
cec_usda.a723_cmolc.kg	mir_cubist_ossl_na_v1.2	0.17	-0.00	0.96	0.98	6.12
cec_usda.a723_cmolc.kg	nir.neospectra_cubist_ossl_na_v1.2	0.38	-0.03	0.72	0.83	2.52
cec_usda.a723_cmolc.kg	visnir_cubist_ossl_na_v1.2	0.34	-0.02	0.80	0.88	2.94
cf_usda.c236_w.pct	mir_cubist_kssl_na_v1.2	0.89	0.05	0.59	0.72	2.33
cf_usda.c236_w.pct	mir_cubist_ossl_na_v1.2	0.90	0.05	0.58	0.71	2.31
cf_usda.c236_w.pct	nir.neospectra_cubist_ossl_na_v1.2	1.16	0.11	0.33	0.48	2.29
cf_usda.c236_w.pct	visnir_cubist_ossl_na_v1.2	0.67	-0.04	0.33	0.47	1.65
clay.tot_usda.a334_w.pct	mir_cubist_kssl_na_v1.2	3.95	0.02	0.94	0.97	5.69
clay.tot_usda.a334_w.pct	mir_cubist_ossl_na_v1.2	5.64	0.10	0.90	0.94	4.20
clay.tot_usda.a334_w.pct	nir.neospectra_cubist_ossl_na_v1.2	7.30	0.12	0.72	0.83	2.60
clay.tot_usda.a334_w.pct	visnir_cubist_ossl_na_v1.2	6.61	0.27	0.82	0.89	3.02
cu.ext_usda.a1063_mg.kg	mir_cubist_kssl_na_v1.2	0.25	0.00	0.86	0.92	4.25
cu.ext_usda.a1063_mg.kg	mir_cubist_ossl_na_v1.2	0.29	0.01	0.78	0.87	3.23
ec_usda.a364_ds.m	mir_cubist_kssl_na_v1.2	0.32	0.03	0.87	0.92	1.72
ec_usda.a364_ds.m	mir_cubist_ossl_na_v1.2	0.31	0.03	0.86	0.92	1.69
ec_usda.a364_ds.m	nir.neospectra_cubist_ossl_na_v1.2	0.36	0.06	0.55	0.65	0.70
ec_usda.a364_ds.m	visnir_cubist_ossl_na_v1.2	0.12	0.02	0.62	0.73	1.28
fe.dith_usda.a66_w.pct	mir_cubist_kssl_na_v1.2	0.15	0.00	0.91	0.95	4.40
fe.dith_usda.a66_w.pct	mir_cubist_ossl_na_v1.2	0.15	0.00	0.91	0.95	4.48

Continuation of S5 Table

Soil property	Model	RMSE	bias	R^2	CCC	RPIQ
fe.dith_usda.a66_w.pct	nir.neospectra_cubist_ossl_na_v1.2	0.25	-0.00	0.62	0.75	2.15
fe.ext_usda.a1064_mg.kg	mir_cubist_kssl_na_v1.2	0.42	0.01	0.80	0.89	2.60
fe.ext_usda.a1064_mg.kg	mir_cubist_ossl_na_v1.2	0.39	0.00	0.76	0.85	2.38
fe.ox_usda.a60_w.pct	mir_cubist_kssl_na_v1.2	0.12	0.01	0.82	0.89	2.75
fe.ox_usda.a60_w.pct	mir_cubist_ossl_na_v1.2	0.13	0.01	0.80	0.88	2.65
fe.ox_usda.a60_w.pct	nir.neospectra_cubist_ossl_na_v1.2	0.15	0.01	0.58	0.70	1.75
k.ext_usda.a1065_mg.kg	mir_cubist_kssl_na_v1.2	0.52	-0.01	0.81	0.88	2.14
k.ext_usda.a1065_mg.kg	mir_cubist_ossl_na_v1.2	0.52	-0.01	0.76	0.85	2.53
k.ext_usda.a725_cmolc.kg	mir_cubist_kssl_na_v1.2	0.17	0.01	0.80	0.88	2.40
k.ext_usda.a725_cmolc.kg	mir_cubist_ossl_na_v1.2	0.17	0.01	0.79	0.87	2.36
k.ext_usda.a725_cmolc.kg	nir.neospectra_cubist_ossl_na_v1.2	0.22	0.03	0.54	0.68	1.75
k.ext_usda.a725_cmolc.kg	visnir_cubist_ossl_na_v1.2	0.23	0.02	0.64	0.76	2.16
mg.ext_usda.a1066_mg.kg	mir_cubist_kssl_na_v1.2	0.47	-0.02	0.90	0.94	4.45
mg.ext_usda.a1066_mg.kg	mir_cubist_ossl_na_v1.2	0.44	-0.03	0.89	0.94	4.39
mg.ext_usda.a724_cmolc.kg	mir_cubist_kssl_na_v1.2	0.26	-0.00	0.92	0.96	5.28
mg.ext_usda.a724_cmolc.kg	mir_cubist_ossl_na_v1.2	0.26	-0.01	0.92	0.96	5.29
mg.ext_usda.a724_cmolc.kg	nir.neospectra_cubist_ossl_na_v1.2	0.41	0.01	0.72	0.83	2.85
mg.ext_usda.a724_cmolc.kg	visnir_cubist_ossl_na_v1.2	0.40	0.01	0.78	0.87	3.27
mn.ext_usda.a1067_mg.kg	mir_cubist_kssl_na_v1.2	0.61	-0.03	0.80	0.88	2.85
mn.ext_usda.a1067_mg.kg	mir_cubist_ossl_na_v1.2	0.68	-0.03	0.76	0.85	2.74
mn.ext_usda.a70_mg.kg	mir_cubist_kssl_na_v1.2	0.74	0.05	0.69	0.79	2.06
mn.ext_usda.a70_mg.kg	mir_cubist_ossl_na_v1.2	0.74	0.06	0.69	0.79	2.06
mn.ext_usda.a70_mg.kg	nir.neospectra_cubist_ossl_na_v1.2	0.78	0.09	0.30	0.42	1.53
n.tot_usda.a623_w.pct	mir_cubist_kssl_na_v1.2	0.06	0.00	0.97	0.98	3.82
n.tot_usda.a623_w.pct	mir_cubist_ossl_na_v1.2	0.06	0.00	0.97	0.98	3.62
n.tot_usda.a623_w.pct	nir.neospectra_cubist_ossl_na_v1.2	0.07	0.00	0.69	0.81	2.01
n.tot_usda.a623_w.pct	visnir_cubist_kssl_na_v1.2	0.10	-0.00	0.93	0.97	5.29
n.tot_usda.a623_w.pct	visnir_cubist_ossl_na_v1.2	0.08	0.00	0.92	0.96	2.52
na.ext_usda.a1068_mg.kg	mir_cubist_kssl_na_v1.2	0.78	0.03	0.75	0.85	2.64

Continuation of S5 Table

Soil property	Model	RMSE	bias	R^2	CCC	RPIQ
na.ext_usda.a1068_mg.kg	mir_cubist_ossl_na_v1.2	0.72	0.02	0.68	0.79	1.66
na.ext_usda.a726_cmolc.kg	mir_cubist_kssl_na_v1.2	0.40	0.03	0.82	0.89	0.48
na.ext_usda.a726_cmolc.kg	mir_cubist_ossl_na_v1.2	0.42	0.04	0.80	0.87	0.47
na.ext_usda.a726_cmolc.kg	nir.neospectra_cubist_ossl_na_v1.2	0.43	0.06	0.42	0.42	0.11
na.ext_usda.a726_cmolc.kg	visnir_cubist_ossl_na_v1.2	0.34	0.04	0.54	0.65	0.78
oc_usda.c729_w.pct	mir_cubist_kssl_na_v1.2	0.11	0.00	0.99	0.99	11.01
oc_usda.c729_w.pct	mir_cubist_ossl_na_v1.2	0.12	0.00	0.99	0.99	9.98
oc_usda.c729_w.pct	nir.neospectra_cubist_ossl_na_v1.2	0.25	-0.01	0.81	0.89	3.22
oc_usda.c729_w.pct	visnir_cubist_kssl_na_v1.2	0.23	-0.01	0.97	0.99	11.24
oc_usda.c729_w.pct	visnir_cubist_ossl_na_v1.2	0.24	-0.00	0.94	0.97	4.20
p.ext_usda.a1070_mg.kg	mir_cubist_kssl_na_v1.2	0.81	0.01	0.68	0.79	2.84
p.ext_usda.a1070_mg.kg	mir_cubist_ossl_na_v1.2	0.80	0.01	0.67	0.79	2.79
p.ext_usda.a1070_mg.kg	nir.neospectra_cubist_ossl_na_v1.2	1.16	-0.01	0.30	0.45	1.80
p.ext_usda.a270_mg.kg	mir_cubist_kssl_na_v1.2	0.76	0.03	0.73	0.83	3.02
p.ext_usda.a270_mg.kg	mir_cubist_ossl_na_v1.2	0.76	0.04	0.72	0.82	2.97
p.ext_usda.a274_mg.kg	mir_cubist_kssl_na_v1.2	0.70	0.02	0.64	0.76	2.59
p.ext_usda.a274_mg.kg	mir_cubist_ossl_na_v1.2	0.74	0.02	0.61	0.74	2.49
p.ext_usda.a274_mg.kg	visnir_cubist_ossl_na_v1.2	1.02	-0.14	0.42	0.55	1.27
ph.cacl2_usda.a481_index	mir_cubist_kssl_na_v1.2	0.36	0.01	0.93	0.96	6.74
ph.cacl2_usda.a481_index	mir_cubist_ossl_na_v1.2	0.36	0.01	0.93	0.96	6.73
ph.cacl2_usda.a481_index	nir.neospectra_cubist_ossl_na_v1.2	0.68	0.02	0.74	0.85	3.54
ph.cacl2_usda.a481_index	visnir_cubist_ossl_na_v1.2	0.38	0.00	0.93	0.96	7.15
ph.h2o_usda.a268_index	mir_cubist_kssl_na_v1.2	0.39	0.00	0.92	0.95	5.83
ph.h2o_usda.a268_index	mir_cubist_ossl_na_v1.2	0.40	0.01	0.91	0.95	5.63
ph.h2o_usda.a268_index	nir.neospectra_cubist_ossl_na_v1.2	0.64	0.01	0.75	0.85	3.39
ph.h2o_usda.a268_index	visnir_cubist_ossl_na_v1.2	0.43	-0.00	0.90	0.95	5.76
s.tot_usda.a624_w.pct	mir_cubist_kssl_na_v1.2	0.07	0.01	0.93	0.96	0.64
s.tot_usda.a624_w.pct	mir_cubist_ossl_na_v1.2	0.07	0.01	0.93	0.96	0.63
s.tot_usda.a624_w.pct	nir.neospectra_cubist_ossl_na_v1.2	0.08	0.01	0.89	0.94	0.34

Continuation of S5 Table

Soil property	Model	RMSE	bias	R^2	CCC	RPIQ
s.tot_usda.a624_w.pct	visnir_cubist_kssl_na_v1.2	0.08	0.01	0.77	0.84	0.47
s.tot_usda.a624_w.pct	visnir_cubist_ossl_na_v1.2	0.08	0.01	0.75	0.83	0.45
sand.tot_usda.c60_w.pct	mir_cubist_kssl_na_v1.2	7.48	0.01	0.93	0.96	6.53
sand.tot_usda.c60_w.pct	mir_cubist_ossl_na_v1.2	8.33	0.00	0.92	0.96	5.84
sand.tot_usda.c60_w.pct	nir.neospectra_cubist_ossl_na_v1.2	18.16	0.63	0.59	0.72	2.58
sand.tot_usda.c60_w.pct	visnir_cubist_ossl_na_v1.2	15.12	0.04	0.68	0.80	2.84
silt.tot_usda.c62_w.pct	mir_cubist_kssl_na_v1.2	6.63	0.05	0.90	0.94	4.60
silt.tot_usda.c62_w.pct	mir_cubist_ossl_na_v1.2	7.31	0.05	0.88	0.93	4.40
silt.tot_usda.c62_w.pct	nir.neospectra_cubist_ossl_na_v1.2	13.79	0.18	0.54	0.67	2.17
silt.tot_usda.c62_w.pct	visnir_cubist_ossl_na_v1.2	11.25	0.16	0.67	0.79	2.49
wr.1500kPa_usda.a417_w.pct	mir_cubist_kssl_na_v1.2	0.18	-0.00	0.94	0.97	4.46
wr.1500kPa_usda.a417_w.pct	mir_cubist_ossl_na_v1.2	0.19	-0.00	0.94	0.97	4.41
wr.1500kPa_usda.a417_w.pct	nir.neospectra_cubist_ossl_na_v1.2	0.36	-0.02	0.65	0.78	2.13
wr.1500kPa_usda.a417_w.pct	visnir_cubist_ossl_na_v1.2	0.43	-0.04	0.61	0.73	1.85
wr.33kPa_usda.a415_w.pct	mir_cubist_kssl_na_v1.2	0.22	0.00	0.83	0.91	2.26
wr.33kPa_usda.a415_w.pct	mir_cubist_ossl_na_v1.2	0.22	-0.00	0.83	0.90	2.27
wr.33kPa_usda.a415_w.pct	nir.neospectra_cubist_ossl_na_v1.2	0.38	-0.03	0.31	0.46	1.21
wr.33kPa_usda.a415_w.pct	visnir_cubist_ossl_na_v1.2	0.35	-0.04	0.57	0.70	1.66
zn.ext_usda.a1073_mg.kg	mir_cubist_kssl_na_v1.2	0.36	0.02	0.74	0.83	2.47
zn.ext_usda.a1073_mg.kg	mir_cubist_ossl_na_v1.2	0.35	0.03	0.60	0.72	1.73
