# Supplementary material for: Open Soil Spectral Library (OSSL): Building reproducible soil calibration models through open development and community engagement
Source: PLoS One. 2025 Jan 13;20(1):e0296545. doi: 10.1371/journal.pone.0296545 (PMC11730021; doi:10.1371/journal.pone.0296545)
Supplement: S3 Table — Statistics are reported for a dataset representing long-term research (LTR) trial sites. Metrics provided in original units after back-transformation. (PDF) [file pone.0296545.s003.pdf]

Supporting Information of *Open Soil Spectral Library (OSSL): Building reproducible soil calibration models through open development and community engagement*

José L. Safanelli[1], Tomislav Hengl[2], Leandro L. Parente[2], Robert Minarik[2], Dellena E. Bloom[3], Katherine Todd-Brown[3], Asa Gholizadeh[4], Wanderson de Sousa Mendes[5], Jonathan Sanderman[1*]

1 Woodwell Climate Research Center, Falmouth, MA, USA

2 OpenGeoHub foundation, Wageningen, the Netherlands

3 University of Florida, Gainesville, FL, USA

4 Czech University of Life Sciences Prague, Czech Republic

5 The Food and Agriculture Organization of the United Nations, Rome, Italy

* Corresponding author. E-mail: jsanderman@woodwellclimate.org

S6 Table. Goodness-of-fit metrics of organic carbon (oc_usda.c729_w.pct) from an independent evaluation of the mid-infrared (MIR) models calibrated with the Open Soil Spectral Library (OSSL) database. Statistics are reported for a dataset representing long-term research (LTR) trial sites. Metrics provided in original units after back-transformation.

Source	Model type	Model subset	n	RMSE	bias	R^2	CCC	RPIQ
KSSL spectra	plsr	kssl	162	0.38	-0.22	0.95	0.92	2.05
KSSL spectra	plsr	ossl	162	0.36	-0.21	0.95	0.93	2.18
KSSL spectra	cubist	kssl	162	0.27	-0.16	0.95	0.95	2.91
KSSL spectra	cubist	ossl	162	0.22	-0.10	0.96	0.97	3.53
Woodwell spectra	plsr	kssl	162	0.44	-0.18	0.92	0.90	1.79
Woodwell spectra	plsr	ossl	162	0.45	-0.22	0.92	0.90	1.74
Woodwell spectra	cubist	kssl	162	0.51	-0.32	0.91	0.87	1.54
Woodwell spectra	cubist	ossl	162	0.24	-0.06	0.94	0.96	3.24
