# Supplementary figures and images for: Open Soil Spectral Library (OSSL): Building reproducible soil calibration models through open development and community engagement
Source: PLoS One. 2025 Jan 13;20(1):e0296545. doi: 10.1371/journal.pone.0296545 (PMC11730021; doi:10.1371/journal.pone.0296545)

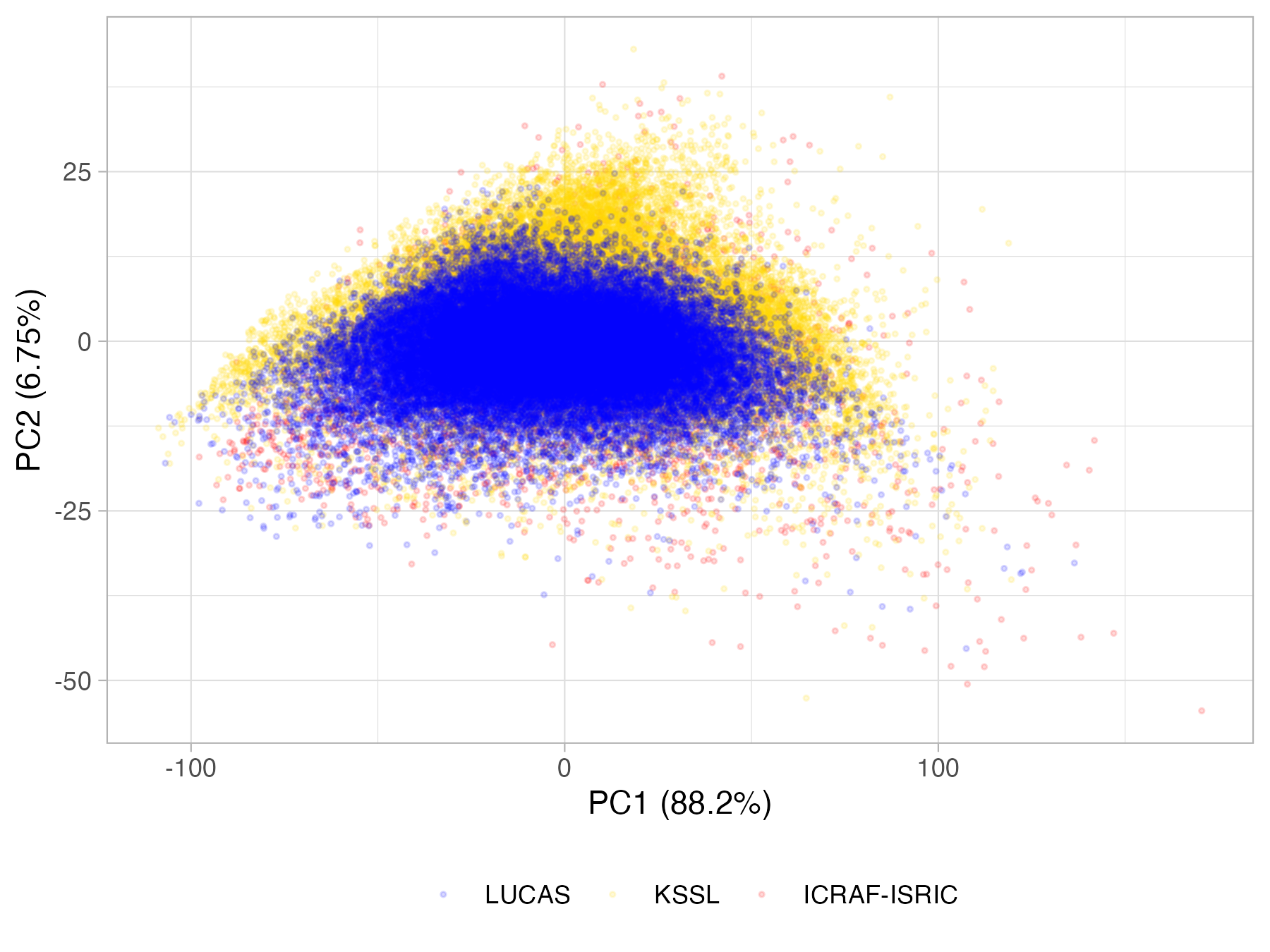

Supplement: S1 Fig — (TIF) [file pone.0296545.s004.tif]

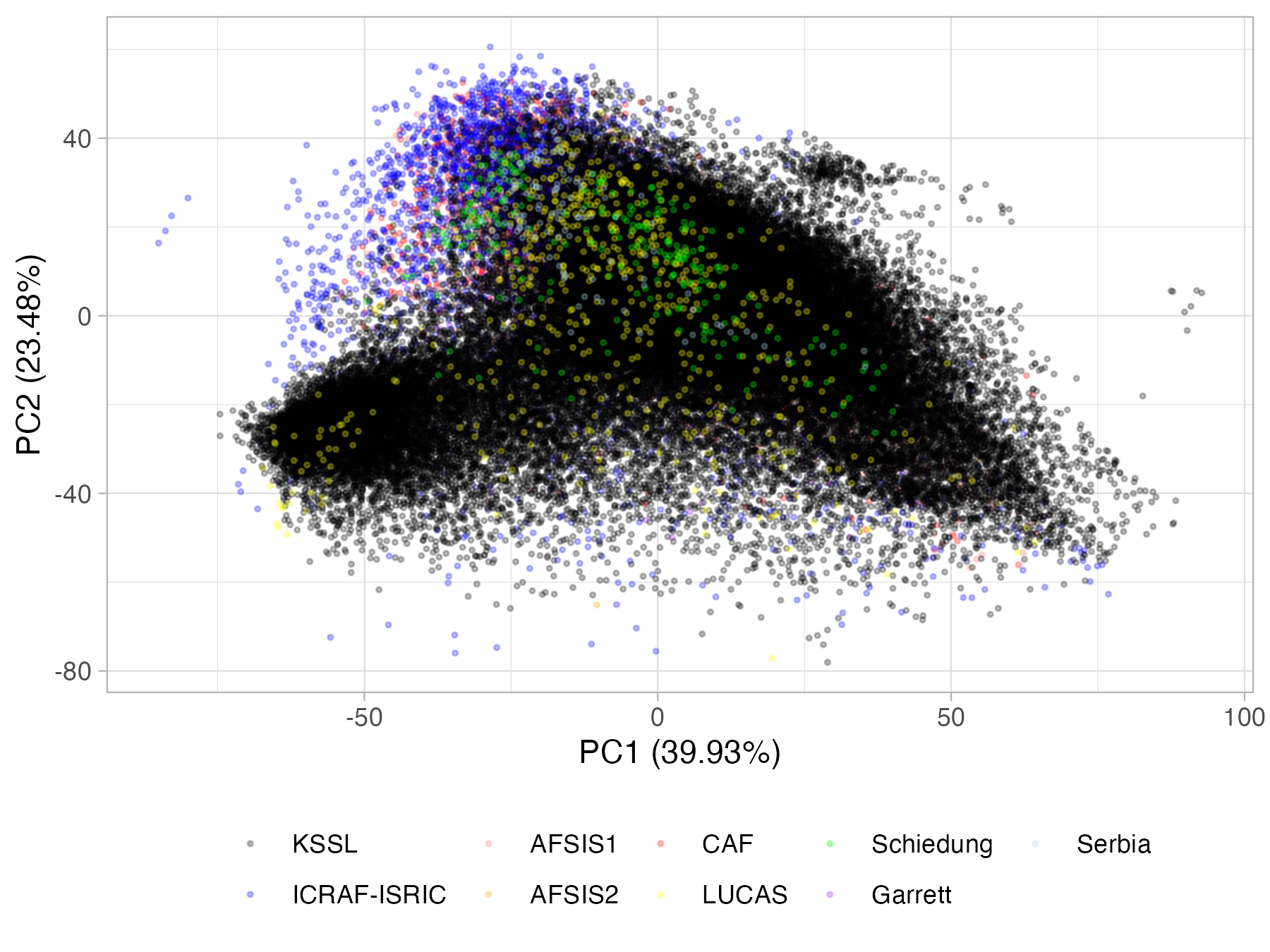

Supplement: S2 Fig — (TIF) [file pone.0296545.s005.tif]

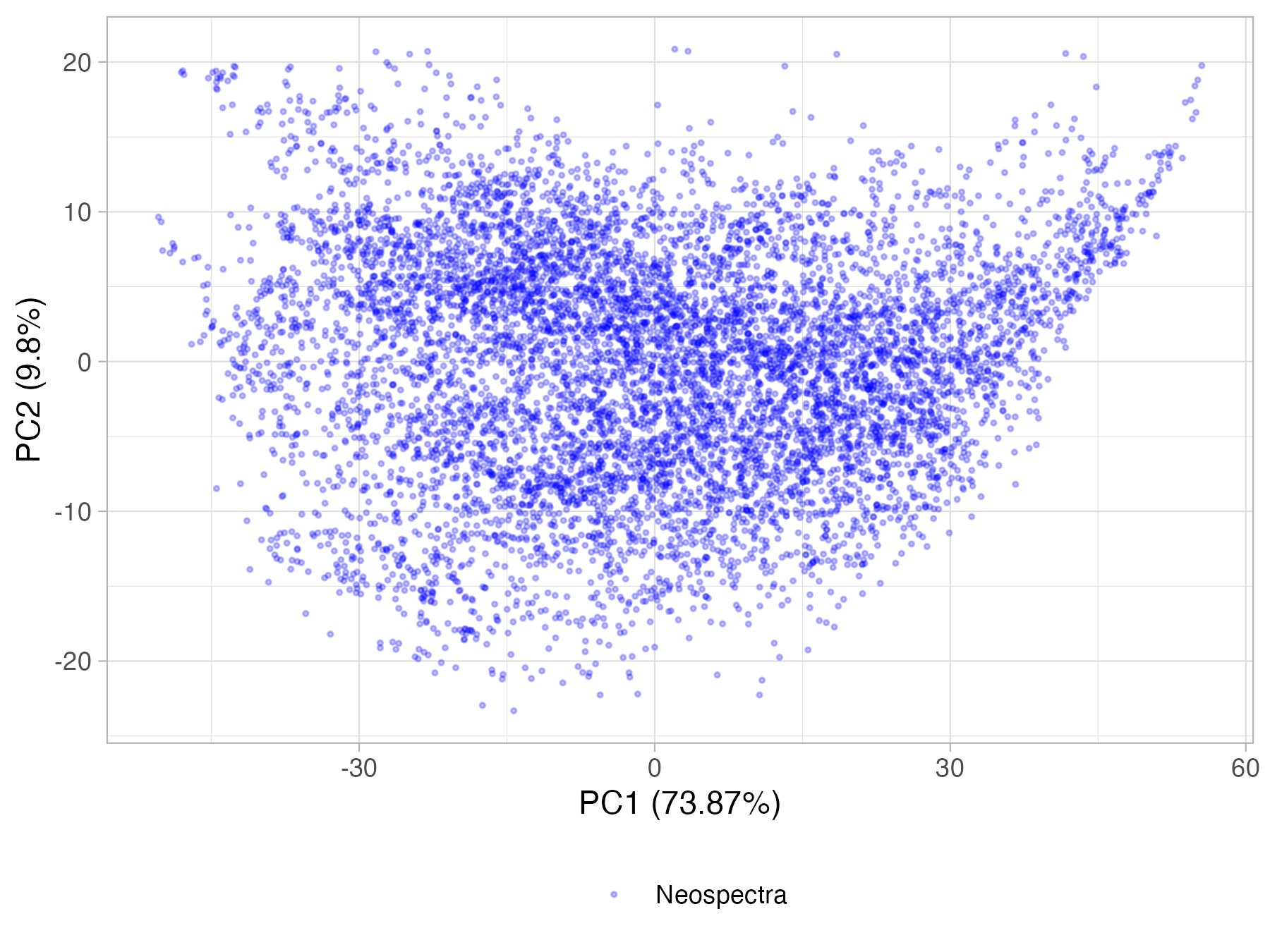

Supplement: S3 Fig — (TIF) [file pone.0296545.s006.tif]
